# Supplementary material for: Adaptation of the Marine Bacterium Shewanella baltica to Low Temperature Stress
Source: Int J Mol Sci. 2020 Jun 18;21(12):4338. doi: 10.3390/ijms21124338 (PMC7352654; doi:10.3390/ijms21124338)
Supplement: Supplementary file 1 [file ijms-21-04338-s001.zip › Supplementary_figures.docx]

SUPPLEMENTARY FIGURES

Supplementary figures for article

Adaptation of the Marine Bacterium
*Shewanella baltica* to Low Temperature Stress

Anna Kloska ^1,^*^,#^, Grzegorz M. Cech ^2^, Marta Sadowska ^2^, Klaudyna Krause ^2^, Agnieszka Szalewska-Pałasz ^2^ and Paweł Olszewski ^3,^*^,#^

^1^ Department of Medical Biology and Genetics, Faculty of Biology, University of Gdańsk, Wita Stwosza 59, 80-308 Gdańsk, Poland

^2^ Department of Bacterial Molecular Genetics, Faculty of Biology, University of Gdańsk, Wita Stwosza 59, 80-308 Gdańsk , Poland

^3^ 3P Medicine Laboratory, International Research Agenda, Medical University of Gdańsk, Dębinki 7, 80-211 Gdańsk, Poland

***** Correspondence: [anna.kloska@ug.edu.pl](mailto:anna.kloska@ug.edu.pl) (A.K.), [pawel.olszewski@gumed.edu.pl](mailto:pawel.olszewski@gumed.edu.pl) (P.O.)

**^#^** Equal contribution to this work


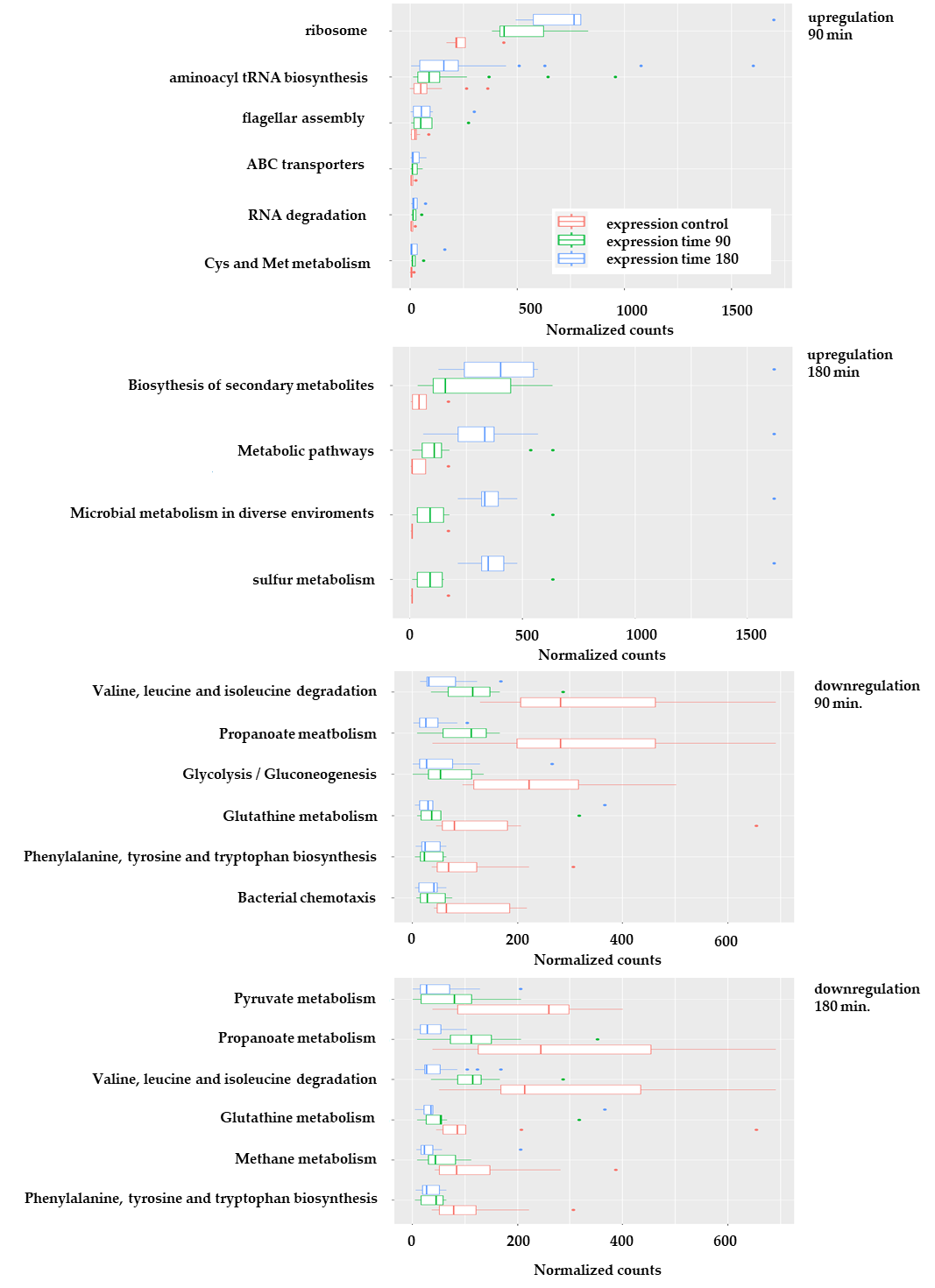


**Figure S1.** Gene enrichment of up- and downregulated metabolic pathways upon cold stress in *S. baltica.* Box-plots show gene enrichment (as normalized counts) at 0, 90 and 180 min of cold shock exposure.


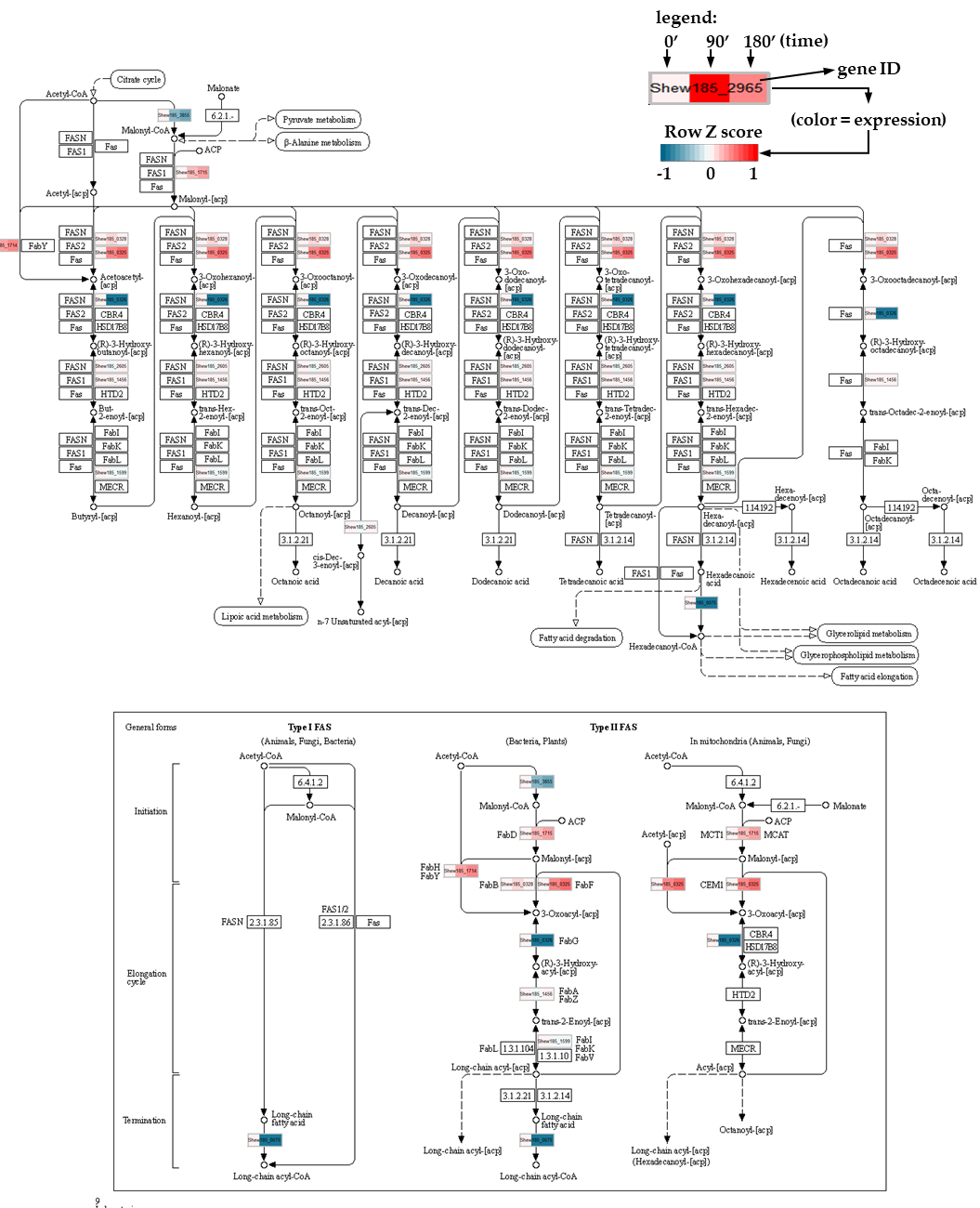


**Figure S2.** Fatty acid biosynthesis metabolic pathways regulated upon cold stress in *S. baltica*. Based on KEGG metabolic pathway maps (Kanehisa Laboratories).


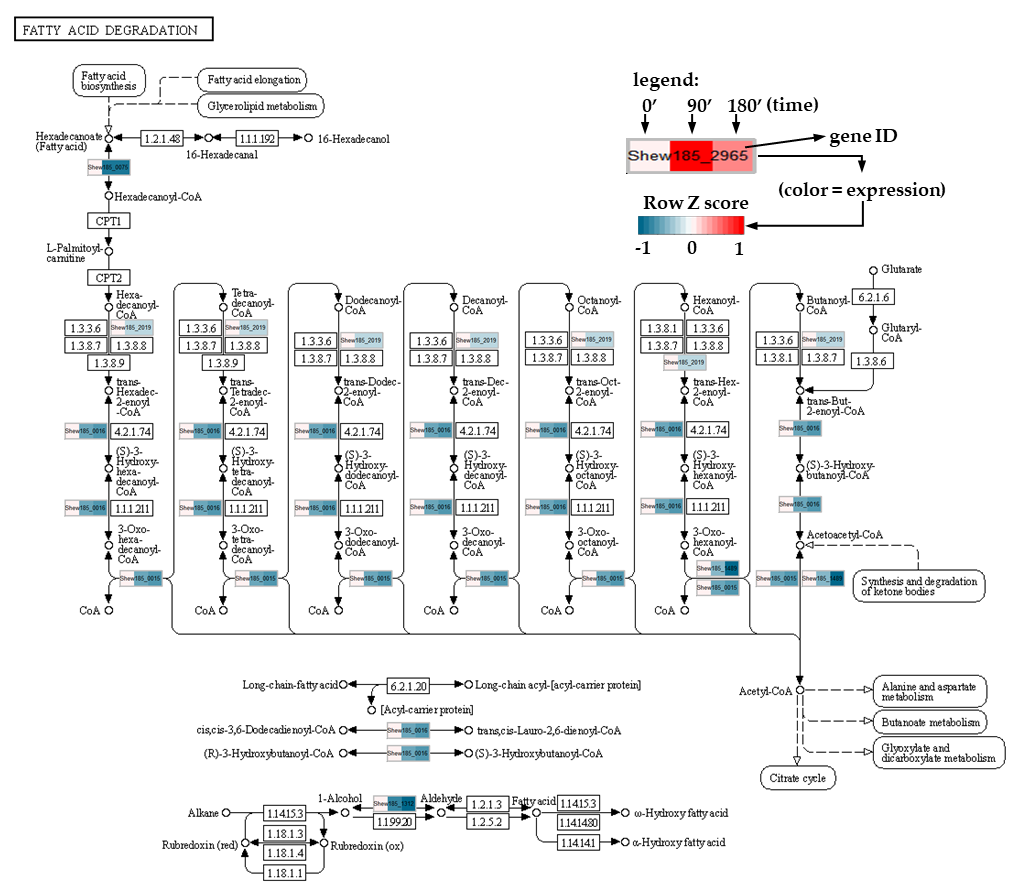


**Figure S3.** Fatty acid degradation metabolic pathway regulated upon cold stress in *S. baltica*. Based on KEGG metabolic pathway maps (Kanehisa Laboratories).
