# Supplementary material for: Adaptation of the Marine Bacterium Shewanella baltica to Low Temperature Stress
Source: Int J Mol Sci. 2020 Jun 18;21(12):4338. doi: 10.3390/ijms21124338 (PMC7352654; doi:10.3390/ijms21124338)
Supplement: Supplementary file 1 [file ijms-21-04338-s001.zip › TableS4_RT-qPCR_primer_list.docx]

**Table S4.** Primers used in RT-qPCR assay. Descriptions of PCR product length, locus tag, gene symbol and protein product are shown.

| **Primer name** | **Primer sequence**  **5’ 🡪 3’** | **PCR product [bp]** | **Locus tag/Gene**  ***S. baltica* OS185** | **Locus tag**  ***S. baltica* M1** | **Protein product** |
| --- | --- | --- | --- | --- | --- |
| **Target genes** | | | | | |
| 2636 | GATCAGTGAGTGTGTCTT  GTGACGACTTGGGTATTT | 142 | Shew185_2636  *rpoE2* | A1L58_14955 | ECF subfamily RNA polymerase sigma-24 factor |
| 1464 | AGCTTGAGGACCTTTTTGG  TGACAATGGTGGCGCTGA | 124 | Shew185_1464 | A1L58_RS06945 | Cold-shock DNA-binding domain-containing protein; CspA family; CspC |
| 2593 | TGCTTAATGGTCGGGAAA  AAGGTATGGGAATTGGTG | 190 | Shew185_2593 | A1L58_21070 | Lysine exporter protein LysE/YggA |
| 3128 | TTTCAGCACCCACATCTT  GGCGCTGTTGGATATTTT | 188 | Shew185_3128 | A1L58_12880 | RNA polymerase sigma factor RpoS; RpoD family |
| M1_HUp | TAGGTACAGTCGCTTCAG  AGCAGCCATTACCGAATC | 146 | Shew185_3817 | A1L58_19690 | Histone family protein DNA-binding protein |
| M1_FlgF | GATGGCAGTGAAGCCTATAC  AGGAAGTGGCAACACTATGG | 120 | Shew185_2958  *flgF* | A1L58_08170 | Flagellar basal body rod protein FlgF |
| M1_RpoE | CGCACAGGAAGCGTTTATC  TCATTAGCGGGCGTTCTAC | 144 | Shew185_1239  *rpoE* | A1L58_05545 | RNA polymerase sigma factor RpoE |
| M1_GroES | CTTCCGACAGGATCAAGACTTC  AATCCTTGCTGTGGGCAATG | 149 | Shew185_0644  *groES* | A1L58_17885 | Co-chaperonin GroES |
| M1_DnaK | GTCGCTTGACGTTGTGAATC  GGCTCCACCACAAGTTTCTG | 132 | Shew185_3411  *dnaK* | A1L58_03265 | Molecular chaperone DnaK |
| M1_RpoD | ATCGCTTGTGGGTATTCG  GTACCGTTGAGCTTCTGAC | 106 | Shew185_1188 | A1L58_05275 | RNA polymerase sigma-70 subunit RpoD |
| M1_FliA | CACGCGACACAGAAATTG  TCCTGAGATACGCCTAAG | 118 | Shew185_2919  *fliA* | A1L58_08380 | Flagellar biosynthesis sigma factor; RNA polymerase sigma factor FliA |
| **Reference genes** | | | | | |
| gyrA | CACGGCGATGCAGAATAAAG  TGGGCGAGGTGGTATTAAAC | 144 | Shew185_2287  *gyrA* | A1L58_20845 | DNA gyrase subunit A |
| gyrB | ATGCAAGAGCGTGAAGATGG  CACTACGGAAACCGACTAAG | 136 | Shew185_0004  *gyrB* | A1L58_18555 | DNA gyrase subunit B |
| mraZ | CAAGGCCGCATCGTGATTAC  TTACCGTCGAGCTCCACTTC | 176 | Shew185_0392  *mraZ* | A1L58_09610 | Cell division protein MraZ |
| rho | ATAACGCTCGCCTTCTTTCG  TTACGCAGCTCAGACGGTTC | 138 | Shew185_2099  *rho* | A1L58_06985 | Rho termination factor domain-containing protein |
| seqA | CCTTTGGCGATAAGCCAACC  AAAGAGGAAACGCCCTACCG | 147 | Shew185_2145  *seqA* | A1L58_04910 | Replication initiation regulator SeqA |
